# RNA triple helix assembled by the poly(A) tail enhances retrotransposon mobilization by preventing RNA deadenylation

**DOI:** 10.1073/pnas.2510774122

**Published:** 2025-09-26

**Authors:** Hui Li, Ling Wang, Zhen Lei, Anna-Sara Biacsi, Dong-Hoon Jeong, Jungnam Cho

**Affiliations:** ^a^Chinese Academy of Sciences Center for Excellence in Molecular Plant Sciences, Chinese Academy of Sciences, Shanghai 200032, China; ^b^Cancer Research United Kingdom Cambridge Institute, University of Cambridge, Cambridge CB2 0RE, United Kingdom; ^c^Department of Pharmacology and Cancer Biology, Duke University, Durham, NC 27710; ^d^Department of Neuroscience, Yale University, New Haven, CT06510; ^e^Department of Biosciences, Durham University, Durham DH1 3LE, United Kingdom; ^f^Department of Life Science, Hallym University, Chuncheon 24252, South Korea; ^g^Multidisciplinary Genome Institute, Hallym University, Chuncheon 24252, South Korea

**Keywords:** element for nuclear expression, RNA triple helix, long terminal repeat retrotransposon, poly(A) tail, *Arabidopsis thaliana*

## Abstract

Transposable elements (TEs), ubiquitous mobile DNAs, are largely repressed by cellular mechanisms such as epigenetic silencing and RNA decay, yet some retain the ability to transpose. The *Arabidopsis* long terminal repeat retrotransposon *Copia93*, also known as *Evade*, exhibits exceptionally high transpositional activity, but the mechanism underlying its extraordinary mobility remains unclear. Here, we identify an Element for Nuclear Expression (ENE) motif within the 3′ UTR of *Evade* that forms a triple helical RNA structure with the poly(A) tail, shielding the transcript from deadenylation and degradation. Deletion of the ENE motif significantly reduces *Evade* transcript stability, extrachromosomal DNA levels, and de novo insertions. Furthermore, we show that the mRNA deadenylase CCR4a directly binds *Evade* RNA to shorten its poly(A) tail, thereby suppressing transposition. Loss of CCR4a leads to *Evade* upregulation, while additional deadenylases contribute to its repression in the absence of CCR4a. Our findings uncover an RNA-based strategy that enhances transposon stability and mobility, illustrating a coevolutionary arms race between host silencing pathways and TE-encoded structural elements.

## Significance

Transposable elements (TEs) are pieces of DNA that can move within a genome, though cells usually suppress them. In the plant *Arabidopsis*, one active TE called *Copia93*, or *Evade*, remains highly mobile. We found that a region in its RNA, the ENE motif, forms a protective structure with the RNA’s tail, shielding it from degradation. Removing this motif reduces *Evade* RNA stability and ability to move. A cellular enzyme, CCR4a, normally shortens *Evade* RNA tail to suppress its activity. Without CCR4a, *Evade* becomes more active, though other enzymes can help control it. This study reveals how *Evade* uses RNA structures to resist cellular defenses, showing a molecular tug-of-war between mobile DNA and host control systems.

Transposable element (TE, transposon) is a mobile DNA that is present in almost all eukaryotic genomes (1, 2). Mobilization of a TE can be deleterious to the host genome because it can disrupt the coding sequence of a functionally critical gene. In eukaryotes, the adverse effects of transposon is prevented primarily by the epigenetic mechanisms (3–5), for which in plants the small interfering (si) RNA-mediated pathway, known as RNA-directed DNA methylation (RdDM), plays a major role (6, 7). Even active and transcribed TEs, observed in abiotic stress conditions and during the reproductive stage, are suppressed by 21/22nt-siRNAs, produced by the RNA-DEPENDENT RNA POLYMERASE 6 (RDR6)-DICER-LIKE 2 or 4 (DCL2/4) module (7–10). Moreover, we recently suggested that in addition to siRNA-mediated suppression of transposon, RNA deadenylation plays a critical role in limiting TE proliferation, implying that transposons are subject to diverse suppressive mechanisms of the host (11).

Despite the tight control of transposon activity, a handful number of TEs in the *Arabidopsis* genome are still able to transpose (12). One example is a *Copia*-type long terminal repeat (LTR) retrotransposon *Copia93* (or *Evade*), which was first identified in the *methyltransferase 1* (*met1*)-derived epigenetic recombinant inbred lines (13). Active mobilization of *Evade* was also observed in the mutants for *DECREASE IN DNA METHYLATION 1* (*DDM1*) and the siRNA biogenesis factors (13–17). In addition, a recent study also suggested that *Evade* has undergone frequent proliferation during evolution, which is evidenced by a large number of unique insertions in the *Arabidopsis* natural population (18). Such strong mobility of *Evade* retroelement contrasts with most other transposons that are transcriptionally activatable but transpositionally inert. However, how *Evade* overcomes the suppressive mechanisms of the host and achieves strong retrotranspositional activity is largely unknown.

It is well documented that RNA secondary structure plays a critical role in the regulation of retrotransposons and retroviruses (19–21). Among these, the Element for Nuclear Expression (ENE) motif features a repeated U-rich tract in the 3′ UTR and was initially discovered in a PAN long noncoding (lnc) RNA of Kaposi’s sarcoma-associated herpesvirus (KSHV) (22). Subsequently, the ENE sequences were identified in various lncRNAs and mRNAs of diverse viruses and eukaryotes (23–25). Intriguingly, it was previously demonstrated that it forms a triple helical structure by base-pairing with the poly(A) tail and thereby protects the mRNA 3′ end from the deadenylation (24, 26, 27). In addition, an extensive bioinformatic search for the ENE sequence was carried out throughout all available eukaryotic genome reference sequences, and around 200 different motifs were identified, most of which are from plants and fungi (28). Importantly, these ENE motifs are strongly associated with LTR retrotransposons, potentiating its biological relevance in the control of transposons in plants (28).

In this study, we identified an ENE sequence motif in the 3′ UTR of *Evade* retroelement of *Arabidopsis*. In vitro RNA denaturation assay revealed that the ENE motif of *Evade* is indeed able to form a triple helix. Most importantly, the loss of ENE sequence resulted in a strong reduction of the steady-state RNA levels, RNA stability and copy number of neoinsertions of *Evade* retrotransposon. Our finding provides an insight into the biological function of the ENE RNA triplex in the enhancement of transpositional activity of a specific retroelement and serves as an intriguing example of an arms race between the host genome and transposon.

## Results

### ENE RNA Triplex Formation of Evade.

**Fig. 1.**
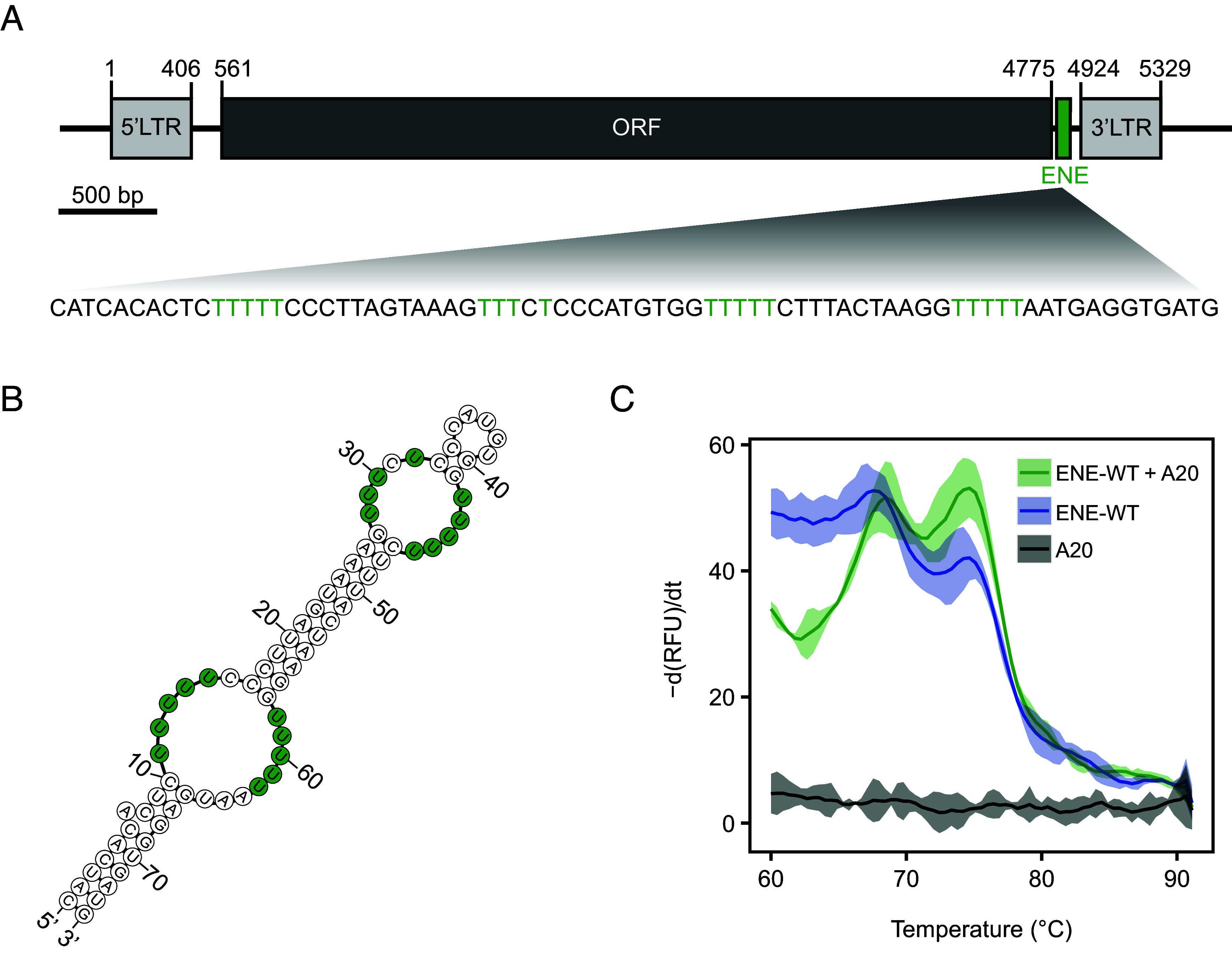
*Evade* contains an ENE RNA triplex motif. (*A*) Schematic illustration of transposon domains and motif organization of *Evade* retrotransposon. Light gray rectangles, long terminal repeat (LTR); dark gray rectangle, open reading frame (ORF); green rectangle, Expression and Nuclear Expression (ENE) motif. Numbers indicate the nucleotide positions, and the U-rich tract of the ENE motif is marked in green. (*B*) Secondary structure of ENE RNA predicted by mfold (http://www.mfold.org/). The U-rich tract is marked in green. (*C*) Thermal denaturation assay (TDA) of ENE motif–containing RNA. Structured RNAs were detected by SYBR Gold in every 0.5 °C of temperature increment. Negative first derivative of relative fluorescence unit (RFU) is plotted. Shaded areas represent 95% CI. ENE-WT, wild-type *Evade* ENE motif–containing RNA; A20, 20-nt A repeat RNA.

Unlike most other TEs in *Arabidopsis* that are transpositionally inactive, *Evade* retrotransposon exhibits exceptionally and unusually strong transposition activity, through mechanisms that are still elusive. While inspecting the *Evade* sequence, we identified a peculiarly long spacer between the ORF and the downstream LTR, a feature that is typically much shorter or even absent in most other retrotransposons. Within this spacer, we found four repeats of unique U-rich tracts, flanked by sequences capable of base-pairing to form a fold-back structure (Fig. 1*A*). RNA secondary structure prediction using mFold software indicated the formation of a stem-loop structure, with the U-rich sequences remaining as internal loops (Fig. 1*B*).

Previous studies in mammals and viruses have proposed that U-rich internal loops can form a triple helical structure with the poly(A) tail through U-A•U interactions (-, Watson–Crick base-pairing; •, Hoogsteen base-pairing), thereby protecting the RNA 3′ end from deadenylation (22, 24, 26, 27, 29). To determine whether the internal loops of *Evade* can form an RNA triple helix, we performed an in vitro thermal denaturation assay (TDA) to assess RNA structural stability across a temperature gradient. The ENE-derived triplex structure is expected to exhibit two distinct dissociation phases: one for the triple helix and another for the stem-loop, producing a biphasic melting curve (*SI Appendix*, Fig. S1). To test this, we allowed in vitro-transcribed ENE RNA from *Evade* to fold into its native secondary structure in the presence and absence of synthetic A-repeat RNAs (denoted as A20), then monitored its strandedness at 0.5 °C temperature increments. Notably, the melting curve of the ENE and A20 RNAs mixture displayed a clear biphasic pattern, whereas the ENE RNA alone showed a significant reduction in the lower-temperature peak which corresponds to the U-rich tract (Fig. 1*C*). These findings strongly indicate that the *Evade* ENE motif can form a triple-helical RNA structure in vitro.

To further characterize the formation of the ENE RNA triplex in *Evade*, we tested variant ENE RNAs, in which U residues within the internal loop were substituted with C, along with mutations introduced in the stem domains (*SI Appendix*, Fig. S2). Mutations 2 and 5, located within the U-rich internal loop, abolished the first transition phase of the melting curve, whereas mutation 7, which disrupted an A-U pair in the stem domain, had no effect on the biphasic pattern (*SI Appendix*, Fig. S2). Additionally, G-C pairs, immediately adjacent to U-rich domains, were essential for triple-helix formation (*SI Appendix*, Fig. S2; mutations 1, 3, 4, and 6), which is consistent with previous reports (24, 26, 29, 30). Together, these results demonstrate that the U-rich domains and their flanking G-C pairs are critical for the formation of the RNA triple helix structure within the *Evade* ENE motif.

### Evade ENE Motif Enhances Gene Expression.

**Fig. 2.**
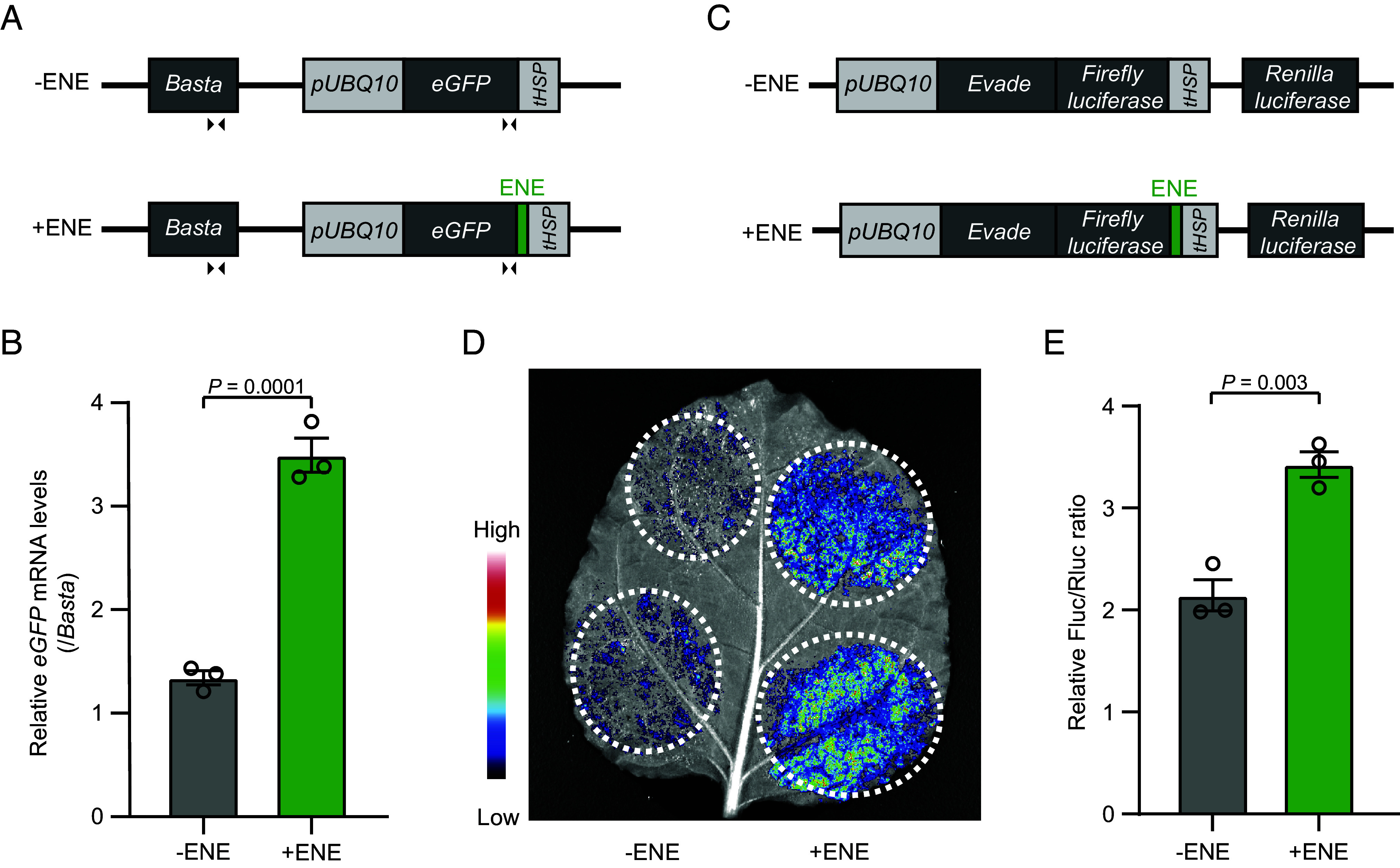
ENE RNA triplex enhances gene expression in plant cells. (*A*) Schematic diagram of the reporter constructs carrying an *eGFP* gene with and without the ENE motif at the 3′ UTR. The arrowheads indicate primers used for RT-qPCR. (*B*) Relative mRNA levels of *eGFP* in tobacco leaves infiltrated with the constructs described in *A*. Normalization was against *BASTA*. Data are mean ± s.e.m of three biological repeats, and the raw data are shown as open circles. A two-sided Welch’s *t* test was used to obtain the *P* value. (*C*) Schematic diagram of the luciferase reporter constructs used for tobacco transient expression experiments. The original *Evade* ORF was inserted upstream of the luciferase gene and the ENE sequence of *Evade* was included in the 3′ UTR. (*D* and *E*) Tobacco transient expression assay of the *Evade*-luciferase recombinant gene with and without the ENE motif. The representative image of a tobacco leaf is shown in *D*. In *E*, the bar plot shows the quantitative measurement of firefly luciferase (Fluc) activity relative to Renilla luciferase (Rluc). Data are mean ± s.e.m of three biological repeats, and the raw data are provided as open circles. A two-sided Welch’s *t* test was used to obtain the *P* value.

Since the ENE RNA triplex is known to stabilize transcripts and enhance gene expression (24, 27, 30), we sought to determine whether the *Evade* ENE motif exerts a similar effect in plants. To test this, we employed a transient expression assay in tobacco, using the commonly used reporter gene *eGFP* with or without the *Evade* ENE motif inserted into the 3′ UTR (Fig. 2*A*). RT-qPCR analysis revealed a significant increase in *eGFP* expression when the ENE motif was present in the 3′ UTR (Fig. 2*B*). A similar effect was observed in a tobacco agroinfiltration assay, in which the *Evade* ORF-containing luciferase gene was expressed with or without the ENE motif (Fig. 2*C*). As shown in Fig. 2 *D* and *E*, the presence of the ENE sequence in the 3′ UTR markedly enhanced luciferase activity. These results strongly suggest that the *Evade* ENE motif promotes gene expression in plant cells presumably by stabilizing transcripts.

### ENE Motif Promotes *Evade* Mobility.

**Fig. 3.**
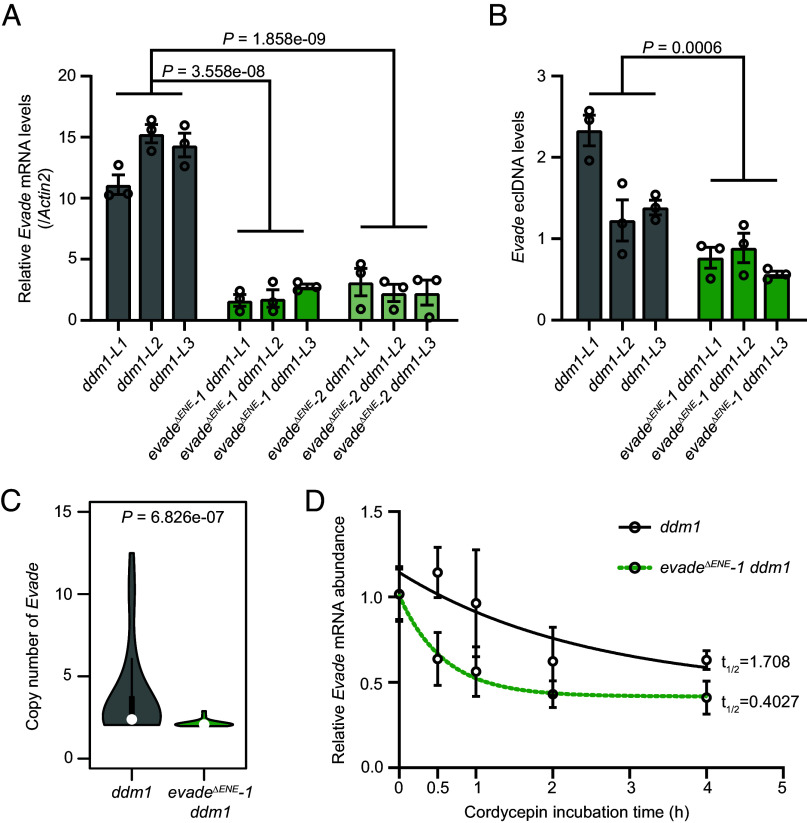
Deletion of the ENE motif leads to reduction of *Evade* transcript stability and mobility. (*A*) *Evade* mRNA levels in the *ddm1*, *evade-1 ddm1,* and *evade-2 ddm1* mutants determined by RT-qPCR. Data are mean ± s.e.m of three biological repeats, and the raw data are shown as open circles. *P* values were obtained by the two-tailed Welch’s *t* test. (*B*) Extrachromosomal linear (ecl) DNA levels of *Evade* in the *ddm1* and *evade-1 ddm1* mutants. Amplification of long terminal repeat of eclDNA (ALE) followed by qPCR was performed to determine the eclDNA levels. PCR-amplified *PopRice* (a rice retrotransposon) DNA was used as spike-in control. Values are mean ± s.d. from three biological replications. *P* values were obtained by the two-tailed Welch’s *t* test. (*C*) *Evade* copy number in *ddm1* (n = 29) and *evade-1 ddm1* (n = 23) mutants at T5 generation, determined by droplet digital PCR (ddPCR). DNA was extracted from individual plants. *P* value was obtained using the two-tailed Wilcoxon rank-sum test. (*D*) mRNA stability of *Evade* in the *ddm1* and *evade-1 ddm1* mutants. Cordycepin was used to arrest transcription and the RNA levels of *Evade* were measured for the time points indicated. The curve was fit to a nonlinear regression model, and t_1/2_ refers to mRNA half-life.

To further investigate the role of the ENE motif in *Evade* retrotransposition, we generated ENE deletion mutants in *Arabidopsis* using CRISPR-Cas9 (*SI Appendix*, Fig. S3). To activate *Evade* transcription, we subsequently induced *ddm1* mutations in both WT and ENE-deficient backgrounds via CRISPR-Cas9 (*SI Appendix*, Fig. S4). Strikingly, the steady-state mRNA levels of *Evade* were markedly reduced in *evade-1 ddm1* and *evade-2 ddm1* double mutants compared to the *ddm1* single mutant (Fig. 3*A*).

To assess whether this reduction affected *Evade* transposition, we measured the levels of extrachromosomal linear (ecl) DNA, a preintegrational reverse-transcribed cDNA intermediate, using the amplification of LTR of eclDNA (ALE)-qPCR method (31). ALE-qPCR revealed a significant decrease in *Evade* eclDNA levels upon ENE deletion (Fig. 3*B*). Consistently, ddPCR analysis showed that the copy number of *Evade* was lower in *evade-1 ddm1* mutants than in *ddm1* single mutants (Fig. 3*C*). These results demonstrate that the ENE motif plays a crucial role in *Evade* transcript stability and retrotranspositional activity.

Given the observed RNA triplex formation and the enhancement of *Evade* expression by the ENE structural motif, we hypothesized that the ENE RNA triplex structure stabilizes *Evade* transcripts, similar to its well-documented role in viruses and mammals. To test this, we conducted an in vivo RNA decay assay to assess *Evade* RNA stability in *evade-1 ddm1* and *ddm1* mutants. Interestingly, ENE deletion led to a significant reduction in *Evade* mRNA stability (Fig. 3*D*). Together, these findings strongly suggest that the ENE motif enhances *Evade* RNA stability by forming an RNA triple helix structure with the poly(A) tail, thereby promoting active retrotransposition.

### mRNA Deadenylase CCR4a Directly Targets *Evade* for Suppression.

**Fig. 4.**
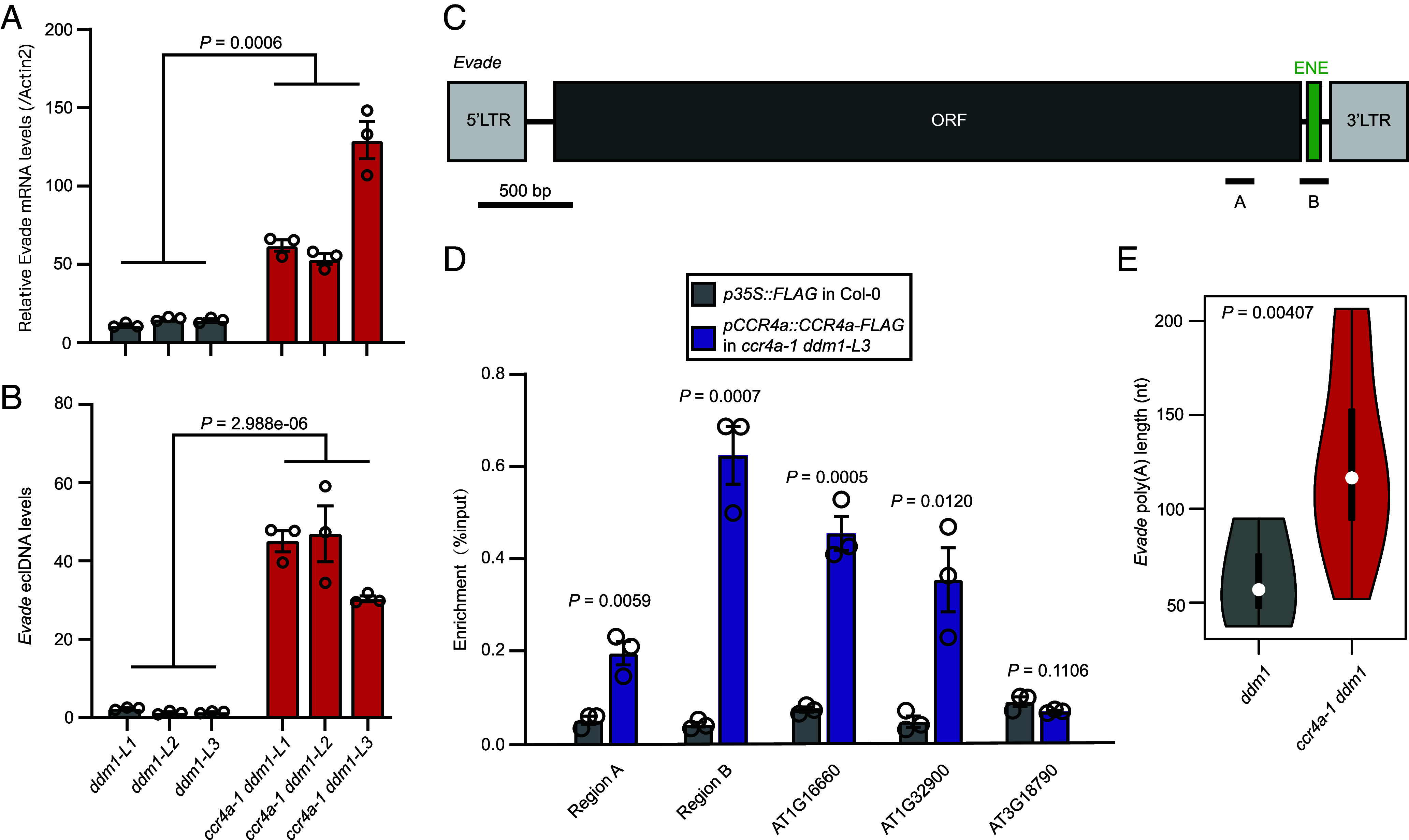
*CCR4a* destabilizes *Evade* transcript by directly binding to it. (*A*) *Evade* mRNA levels in *ddm1* and *ccr4a-1 ddm1* mutants determined by RT-qPCR. Data are shown as mean ± s.e.m. Open circles are individual data of independent biological repeats. *P* value was determined by two-tailed Welch’s *t* test. (*B*) *Evade* eclDNA levels in the *ddm1* and *evade-1 ddm1* mutants. ALE-qPCR was performed to determine the eclDNA levels. PCR-amplified *PopRice* DNA was used as spike-in control. Values are mean ± s.d. from three biological replications. *P* value was obtained by the two-tailed Welch’s *t* test. (*C* and *D*) RNA immunoprecipitation (RIP)-qPCR assay showing the binding affinity of CCR4a protein to *Evade* RNA in vivo. *Evade* regions tested for RIP-qPCR are shown in c. *p35S::FLAG* in Col-0 was used as negative control sample for IP. AT1G16660 and AT1G32900 are positive controls for CCR4a binding, and AT3G18790 a negative control. The binding enrichment was calculated as the ratio of IP to input. Values are mean ± s.d. from three biological replications. *P* values were obtained by the two-tailed Welch’s *t* test. (*E*) Poly(A) tail lengths of *Evade* transcripts in *ddm1* and *ccr4a-1 ddm1* mutants, determined by Oxford Nanopore Technologies direct RNA sequencing. The raw sequencing data were retrieved from PRJNA940263. *P* value was obtained using the two-tailed Wilcoxon rank-sum test.

Our data thus far suggest that *Evade* transposition is regulated at the level of RNA metabolism, specifically through mRNA poly(A) tail dynamics, including deadenylation, a key step preceding mRNA decay (32, 33). In our previous study, we identified the mRNA deadenylase CCR4a as a major factor in shortening transposon RNA tails, thereby suppressing their transposition (11). Notably, the loss of *CCR4a* resulted in the overproliferation of various transposon families, including *Evade* (11). To determine whether CCR4a directly regulates *Evade* RNA stability via poly(A) tail shortening, we generated *ccr4a-1 ddm1* double mutants using CRISPR-Cas9 (*SI Appendix*, Fig. S5). RT-qPCR and ALE-qPCR analyses revealed that mutations in *CCR4a* led to a significant upregulation of *Evade* RNA and eclDNA levels (Fig. 4 *A* and *B*), further supporting our previous findings that CCR4a plays a critical role in repressing *Evade* retrotransposition (11).

**Fig. 5.**
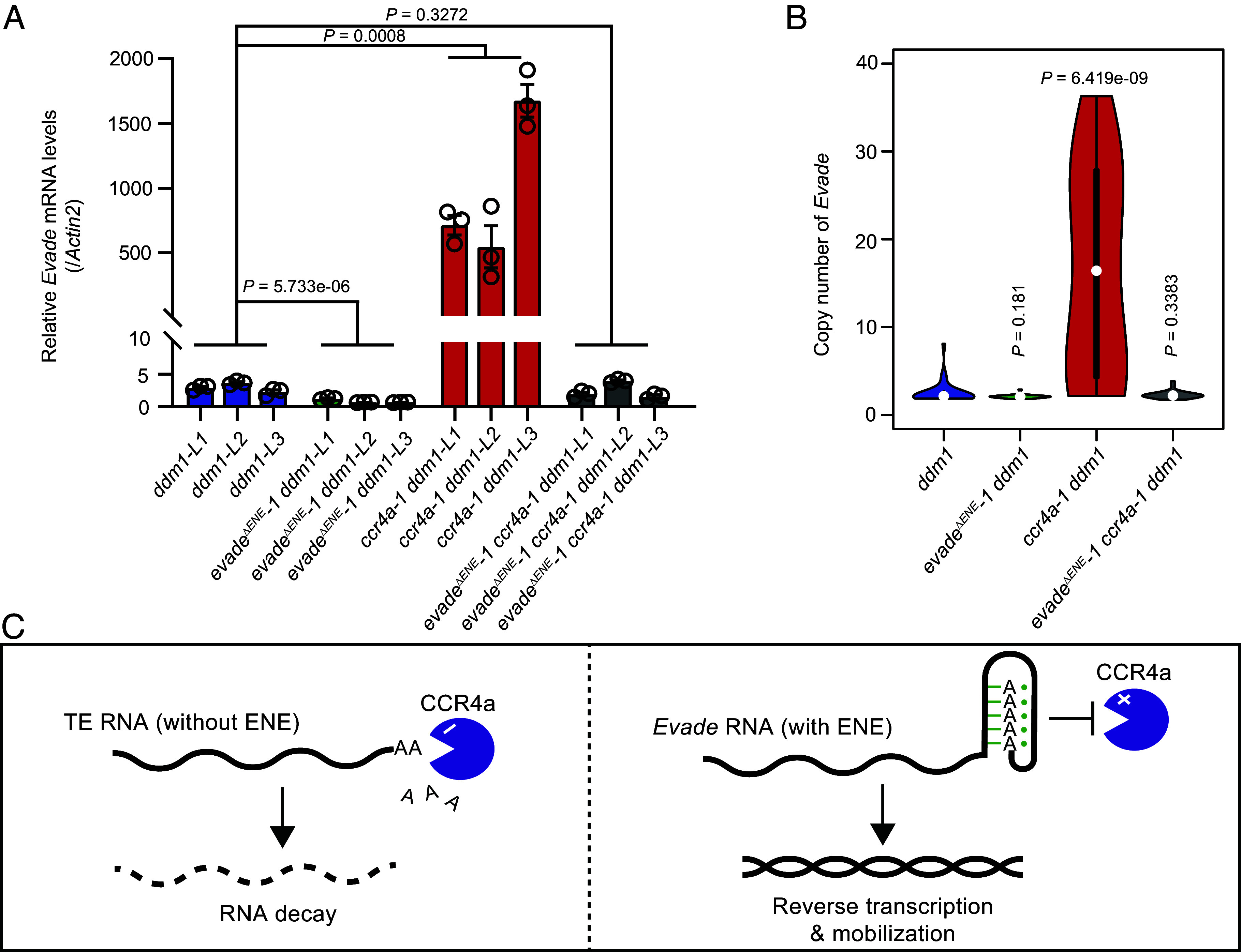
Functional redundancy of various mRNA deadenylases. (*A*) *Evade* mRNA levels in the *ddm1*, *evade-1 ddm1*, *ccr4a-1 ddm1*, and *evade-1 ccr4a-1 ddm1* mutants at T4 generation, determined by RT-qPCR. Data are mean ± s.e.m of three biological repeats, and the raw data are shown as open circles. *P* values were obtained by the two-tailed Welch’s *t* test. (*B*) *Evade* copy number in the *ddm1* (n = 30), *evade-1 ddm1* (n = 29), *ccr4a-1 ddm1* (n = 24), and *evade-1 ccr4a-1 ddm1* (n = 29) at T4 generations, assessed by ddPCR. DNA was extracted from individual plants. *P* values were determined using the two-tailed Wilcoxon rank-sum test. (*C*) A schematic illustration for the role of ENE in *Evade* retrotransposon control. TE RNAs, which lack the ENE motif, are subject to cytoplasmic RNA decay initiated by CCR4a RNA deadenylase. *Evade* retroelement contains a unique U-rich sequences forming an RNA triplex in conjunction with the poly(A) tail, which then protects the transcripts from CCR4a-mediated RNA deadenylation. The enhanced RNA stability of *Evade* by the ENE motif leads to the increased mobility.

We then performed RIP-qPCR using the *Arabidopsis* transgenic plants expressing CCR4a-FLAG in the *ccr4a-1 ddm1* double mutant background (Fig. 4*C*). To validate CCR4a binding specificity, we selected three transcripts as positive and negative controls (*SI Appendix*, Fig. S6). RIP-qPCR analysis revealed a significant enrichment of CCR4a-FLAG on *Evade* RNA, particularly at the 3′ end and in the vicinity of the ENE motif (Fig. 5*D*).

To further investigate the impact of CCR4a on *Evade* RNA stability, we analyzed poly(A) tail lengths using Oxford Nanopore Technologies direct RNA sequencing (ONT-DRS). Compared to *ddm1*, *ccr4a-1 ddm1* mutants exhibited significantly longer poly(A) tails on *Evade* transcripts (Fig. 4*E*). Taken together, these results strongly suggest that the mRNA deadenylase CCR4a directly binds *Evade* RNA and represses its retrotransposition by shortening its poly(A) tail.

To further investigate the genetic relationship between the ENE RNA structural motif and the mRNA deadenylase CCR4a, we generated *evade-1 ccr4a-1 ddm1* triple mutants and examined *Evade* RNA levels and copy number. As shown in Fig. 5, the triple mutants exhibited a strong reduction in *Evade* RNA levels and copy number compared to the *ccr4a-1 ddm1* double mutants. This suggests that additional mRNA deadenylases may compensate for CCR4a’s function in its absence. Supporting this notion, RNA-seq analysis of various mRNA deadenylase mutants revealed a substantial increase in *Evade* RNA levels in *ccr4b* and *caf1a caf1b* double mutants (*SI Appendix*, Fig. S7). These findings indicate that while CCR4a plays a primary role in repressing *Evade* RNA, other deadenylases, such as CCR4b, CAF1a, and CAF1b (34–36), also contribute to *Evade* RNA deadenylation.

## Discussion

Transposons are subject to stringent host surveillance mechanisms that limit their mobility to preserve genome integrity. Despite this, some TEs, such as the *Evade* retrotransposon in *Arabidopsis*, exhibit unusually high transpositional activity. In this study, we identified a ENE RNA structural motif within the 3′ UTR of *Evade* and demonstrated that it plays a crucial role in enhancing transcript stability and transpositional activity. Our findings suggest that *Evade* has evolved a mechanism to evade host suppression through an RNA-based strategy that protects its transcripts from degradation (illustrated in Fig. 5*C*).

Our in vitro RNA denaturation assays revealed that the ENE motif of *Evade* forms a triple helical RNA structure with the poly(A) tail (Fig. 1 and *SI Appendix*, Fig. S2), a mechanism previously described in viruses and mammalian noncoding RNAs (22, 24, 29). The formation of this structure effectively shields *Evade* transcripts from mRNA decay pathways (Fig. 3), providing a selective advantage by extending transcript longevity. This mechanism is consistent with prior reports showing that ENE motifs in other systems enhance RNA stability and translation efficiency (27, 30). Given that *Evade* is one of the most active retrotransposons in *Arabidopsis*, our findings suggest that RNA structural elements can serve as a key regulatory feature enabling persistent TE activity. Notably, *Evade* is the only retroelement that, among many transcribed transposons in *ddm1* mutants, contains the ENE motif at its 3′ UTR. This unique feature may contribute to its exceptional mobility compared to other transcriptionally activated but transpositionally inert elements.

The ability of *Evade* to exploit an ENE-mediated strategy to counteract host repression provides an intriguing example of the ongoing coevolutionary arms race between transposons and their host genomes. While plants have developed robust RNA silencing and degradation mechanisms to suppress transposons, *Evade* appears to have evolved an RNA-based strategy to evade these defenses. Although ENE motifs are seemingly rare in the *Arabidopsis* genome, Tycowski et al. identified similar motifs in transposon RNAs from various plant species (28). This suggests that ENE-mediated transcript stabilization may be more widespread than previously recognized, potentially representing a conserved strategy among TEs to sustain their lifecycle within host genomes.

Our findings open broad avenues for exploring how RNA secondary structures influence transposon biology and host genome evolution. Future studies should investigate whether similar ENE-like motifs exist in other plant transposons and assess their functional significance in TE regulation. Furthermore, given the potential impact of TEs on genome evolution, deciphering the interplay between RNA structural elements and host silencing mechanisms could inform strategies for genome engineering and TE-based applications in biotechnology.

In summary, our study reveals that the ENE RNA triplex of *Evade* serves as a critical determinant of its transpositional activity by stabilizing its transcripts against host-mediated deadenylation and decay. These findings expand our understanding of transposon regulation and illustrate an RNA-based strategy employed by TEs to persist within host genomes.

## Materials and Methods

### Plant Materials and Growth Condition.

All *Arabidopsis* plants used in this study were in the Col-0 background. The mutant seeds (*ccr4a-1*, SAIL_802_A10; *ccr4b-1*, SALK_151541C; *caf1a-1*, SALK_070336; *caf1b-3*, SALK_044043) were obtained from the Arabidopsis Biological Resource Center (ABRC; https://abrc.osu.edu/) and validated for their genotypes as previously described (37). The *evade-1* and *evade-2* mutants were generated by CRISPR-Cas9 targeting the ENE motif of *Evade* retrotransposon. The *evade-1 ccr4a-1* double mutant was generated by crossing the single mutants. *De novo DDM1* mutants were generated by CRISPR-Cas9 in the indicated mutant background. Construction of CRISPR vectors was as previously described (11, 38), and sequences of primers used for genotyping are listed in *SI Appendix* Table S1.

Seeds were surface sterilized in 75% ethanol solution and sown on Murashige and Skoog (MS) media (0.43 g/L MS salts, 3 g/L sucrose, 0.8% agar, pH 5.8). After stratification at 4 °C for 3 d, plants were germinated and grown at 22 °C under long-day conditions (cool-white fluorescent light on for 16 h and off for 8 h). Whole seedlings were used for all experiments.

### TDA.

The *Evade* fragments containing the intact ENE and mutated ENE sequences were prepared by PCR amplification using synthesized oligonucleotides as template. PCR fragments were subsequently used for in vitro transcription by a HiScribe T7 High Yield RNA Synthesis Kit (NEB), and the transcribed RNA was cleaned up by a Monarch® RNA Cleanup Kit (NEB). 3 µM of RNA and A20 (20-nt A repeat of RNA oligonucleotides) was mixed in the 1:1 molar ratio in the 20 µL incubation buffer [10 mM Tris-HCl (pH 7) and 10 mM MgCl_2_]. The mixture was first heated at 95 °C for 55 s and slowly cooled down to room temperature for renaturation. The RNA mixture was then subjected to a slow denaturation by incrementing the temperature from 50 to 95 °C. SYBR Gold (Invitrogen) was used to visualize base-paired and structured RNA. The fluorescence-monitored melting profile was detected using a CFX96 Connect Real-Time PCR Detection System (Bio-Rad). Sequences of *Evade* ENE fragments are listed in *SI Appendix* Table S1.

### RT Followed by qPCR (RT-qPCR).

RT-qPCR experiments were carried out by following the manufacturers’ instructions and as previously described (37). Plant RNA was extracted using the TRIzol solution (TIANGEN). ReverTra Ace qPCR RT Master Mix with gDNA Remover (TOYOBO) was then used to reverse-transcribe the extracted RNA. The resulting cDNA was subjected to quantitative PCR using a ChamQ Universal SYBR qPCR Master Mix (VAZYME) on a CFX96 Connect Real-Time PCR Detection System (Bio-Rad). Normalization was against *Actin2* (AT3G18780) in *Arabidopsis* and *EF-1α* in tobacco. RNA levels were determined by the 2^−ΔΔCt^ method. Sequences of primers used are listed in *SI Appendix* Table S1.

### ddPCR.

The DNA copy number of retrotransposons was assessed by ddPCR, performed on TargetingOne® Digital PCR System (TargetingOne) following the previously described method (39, 40). First, 100 ng of total DNA was digested using the restriction enzyme AluI (NEB). The reaction mixture was set up by adding 2 × ddPCR Supermix (Bio-Rad), 0.8 μM of each primer and 0.25 μM probe, and 0.6 ng of digested DNA. The mixture and 180 μL of droplet generation oil were loaded on the droplet generation chip. The droplets (approximately 50,000 to 60,000 per test) were then transferred to an 8-strip PCR tube, and PCR was performed on a PTC-200 Thermal Cycler (Bio-Rad; 95 °C for 10 min, 55 cycles of denaturation at 95 °C for 30 s and annealing at 56.8 °C for 1 min, enzymatic inactivation at 98 °C for 10 min). After PCR, FAM (488 nm) and HEX (532 nm) fluorescence signals were detected by the Chip Reader. Finally, the number of fluorescent droplets was subjected to Poisson distribution analysis using the Chip Reader R1 software. Sequences of primers and probes are listed in *SI Appendix* Table S1.

### Tobacco Transient Expression.

Tobacco (*Nicotiana benthamiana*) plants were grown on soil in a controlled environment chamber with a day/night cycle of 16 h/8 h at 22 °C. Around 4-wk-old plants were used for agro-infiltration of GV3101 strain together with the helper plasmid p19. Leaf discs were collected after 3 d of agro-infiltration. All tissue samples were collected from three replicate plants. Sequences of primers are listed in *SI Appendix* Table S1.

### Dual-Luciferase (Dual-LUC) Assay.

For dual-LUC assays, *pUBQ10::EVADE-Fluc:tHSP18.2* and *pUBQ10::EVADE-Fluc:ENE-tHSP18.2* was constructed. The Renilla luciferase (Rluc) gene was driven by the constitutive *enTCUP2* promoter and used as an internal reference gene. Plasmids were transformed into Agrobacterium strain GV3101 and infiltrated to tobacco leaves as described above. Leaves were collected after 48 h of infiltration to measure the Fluc and Rluc activities using Dual-Luciferase® Reporter Assay System (Promega). Three biological repeats were performed. Sequences of all primers are listed in *SI Appendix* Table S1.

### Transcription Arrest Profiling.

4-d-old *Arabidopsis* seedlings were placed on a filter paper soaked with the incubation buffer containing cordycepin [1 mM PIPES (pH 6.25), 15 mM sucrose, 1 mM potassium chloride, 1 mM sodium citrate, and 1 mM cordycepin]. To facilitate the absorption of cordycepin into plant tissues, the plants were vacuum infiltrated three times, for 1 min each. Samples for RT-qPCR were harvested at 0, 30, 60, 120, and 240 min.

### Amplification of LTR of eclDNA (ALE)-qPCR.

ALE-qPCR was performed as previously described (31, 41, 42). Briefly, plant total DNA was purified using a Plant Genomic DNA Kit (TIANGEN) following the manufacturer’s instructions. 200 ng of total DNA, along with 1 pg of PCR-amplified *PopRice* DNA, were ligated to 0.5 µL of 40 µM adapter DNA at 16 °C overnight (the sequences are provided in *SI Appendix* Table S1). AMPure XP beads (Beckman Coulter) were used at a 1:0.5 ratio to discard unligated adapters and recover adapter-ligated DNA. Subsequently, T7 in vitro transcription reactions were carried out using a Standard RNA Synthesis Kit (NEB). The synthesized RNA product was purified by a Monarch RNA Cleanup Kit (NEB). cDNA was obtained by reverse-transcribing 1 µg of RNA using a Transcriptor First Strand cDNA Synthesis Kit (Roche). To degrade nontemplated RNA, 1 µL of RNase A/T1 (Thermo Fisher Scientific) was added and incubated for 30 min at 37 °C. Finally, qPCR was performed as described above, and the oligonucleotide sequences are provided in *SI Appendix* Table S1.

### RIP-qPCR.

RIP-qPCR was used to assess the association of a protein to transcripts, following the method previously described (41). Briefly, the plants expressing *pCCR4a::CCR4a-FLAG* in *ccr4a-1 ddm1* and *p35S::FLAG* in Col-0 were grown at 22 °C under long-day condition for 12 d. 2 g of seedlings was harvested, cross-linked for 10 min under a vacuum in 1% formaldehyde solution (Sigma), and quenched by adding glycine to a final concentration of 0.125 M. The plant samples were washed in water, flash frozen in liquid nitrogen, and ground to fine powder. Frozen powder was homogenized in 2 mL of extraction buffer [100 mM Tris-HCl (pH 7.5), 150 mM NaCl, 0.5% IGEPAL (Sigma), 40 U/mL RNase inhibitor (ABclonal), and 1% plant protease inhibitor cocktail (MedChem Express)]. The homogenized sample was rotated at 4 °C for 2 h and then centrifuged for 20 min at 18,000×*g* at 4 °C. The supernatant was recovered, and RNAs were sonicated using the Branson sonifier for 7 × 10 s with 20 s interval at low amplitude, which sheared RNAs to approximately 200 to 400 nt. In 2 mL of the sample, 70 µL anti-FLAG beads (Smart-Lifesciences) were added and incubated overnight at 4 °C. 200 µL of the solution was kept aside for an input sample by storing at −80 °C. After incubation, the beads were washed four times using 1 mL extraction buffer, resuspended in 150 µL of the proteinase K buffer (15 µL 10% SDS, 18 µL 10 mg/mL proteinase K, and 117 µL extraction buffer), and then incubated for 30 min at 55 °C. RNA was then extracted by the TRIzol method (TIANGEN) as described above. The input sample stored at −80 °C was retrieved and extracted for RNA using the same method. The immunoprecipitated and input RNAs were processed for cDNA synthesis and qPCR, following the same procedures as previously described. The oligonucleotide sequences are provided in *SI Appendix* Table S1.

## Supplementary Material

Appendix 01 (PDF)

## Data Availability

The analyses were performed using the standard codes instructed by the tools as indicated in the Methods, and the parameters used for the analyses were as previously described (11). All other data are included in the manuscript and/or *SI Appendix*.
