# Supplementary material for: RNA triple helix assembled by the poly(A) tail enhances retrotransposon mobilization by preventing RNA deadenylation
Source: Proc Natl Acad Sci U S A. 2025 Sep 26;122(39):e2510774122. doi: 10.1073/pnas.2510774122 (PMC12501189; doi:10.1073/pnas.2510774122)
Supplement: Supplementary file 1 — Appendix 01 (PDF) [file pnas.2510774122.sapp.pdf]

PNAS

Supporting Information for

RNA triple helix assembled by poly(A) tail enhances retrotransposon mobilization by preventing RNA deadenylation

Hui Li[1,2], Ling Wang[1,3], Zhen Lei[1,4], Anna-Sara Biacsi[5], Dong-Hoon Jeong[6,7] and Jungnam Cho[5]*

[1]CAS Center for Excellence in Molecular Plant Sciences, Chinese Academy of Sciences, Shanghai 200032, China.
[2]Cancer Research UK Cambridge Institute, University of Cambridge, Cambridge CB2 0RE, United Kingdom.
[3]Department of Pharmacology and Cancer Biology, Duke University, Durham 27710, United States.
[4]Department of Neuroscience, Yale University, New Haven 06510, United States.
[5]Department of Biosciences, Durham University, Durham DH1 3LE, United Kingdom.
[6]Department of Life Science, Hallym University, Chuncheon 24252, South Korea.
[7]Multidisciplinary Genome Institute, Hallym University, Chuncheon 24252, South Korea.
*Corresponding author (Jungnam Cho)

Email: jungnam.cho@durham.ac.uk

This PDF file includes:

Figures S1 to S7
Tables S1

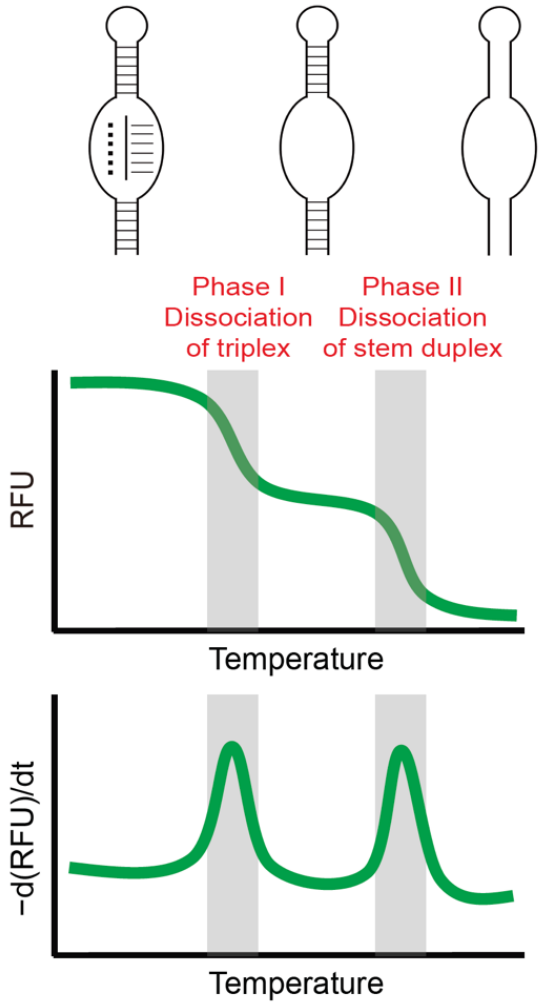

Fig. S1. Thermal denaturation assay. Diagram of fluorescence intensity curve of ENE RNA triplex across temperature increment. The left and right peaks represent the dissociation of triplex and stem duplex, respectively. RFU, relative fluorescence unit.

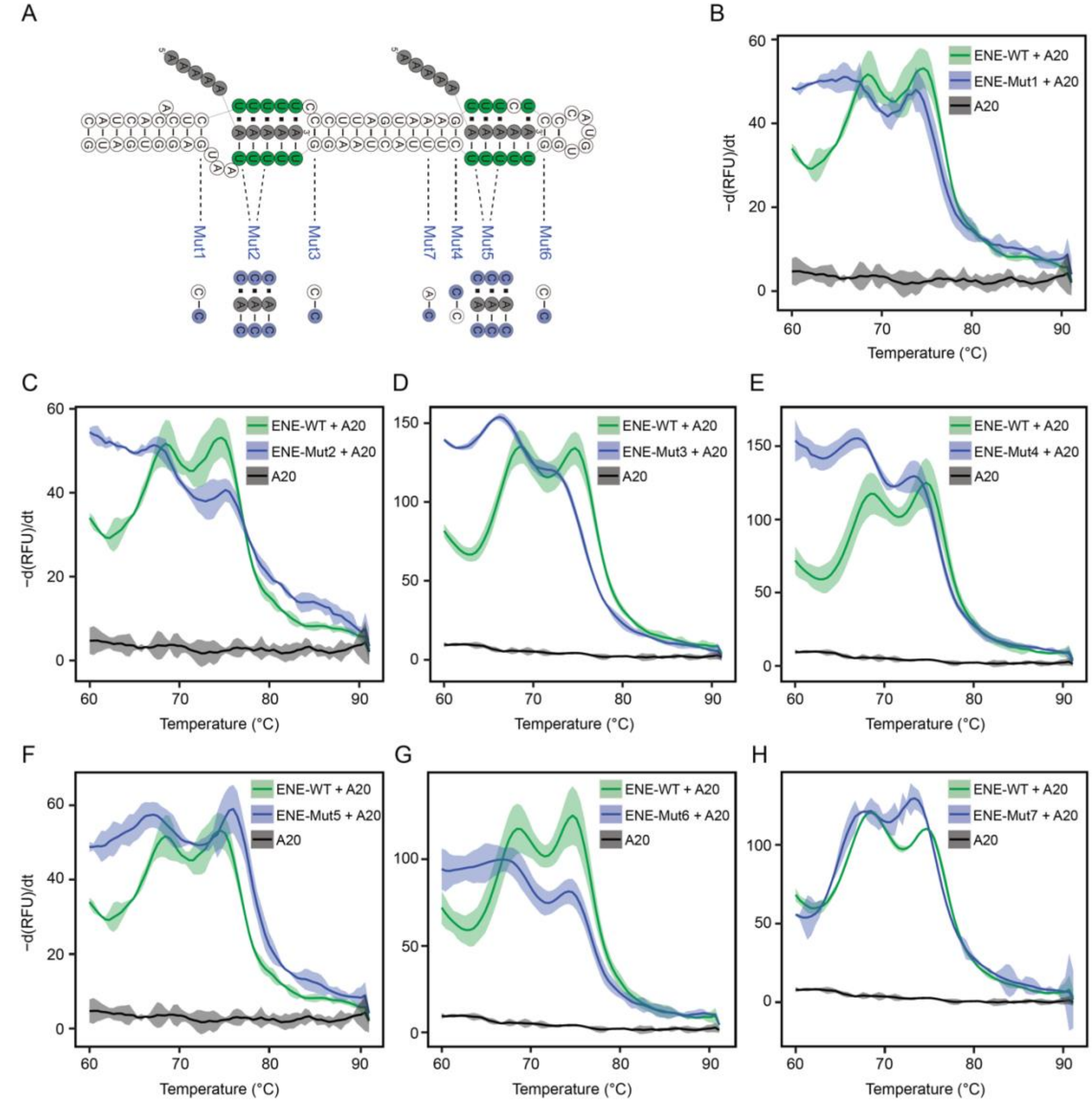

Fig. S2. Site-specific mutations in ENE and their triplex-forming abilities. (A) Schematic diagram of a predicted RNA triplex structure formed by the *Evade* ENE and poly(A) RNA. Green, U-rich tract; grey, poly(A); blue, mutated bases. (B-H) Thermal denaturation assays using the base substituted *Evade* ENE RNAs. Fluorescence of structured RNAs was detected in every 0.5 °C of temperature increment. Negative first derivative of relative fluorescence unit (RFU) is plotted. Shaded areas represent 95% confidence intervals.

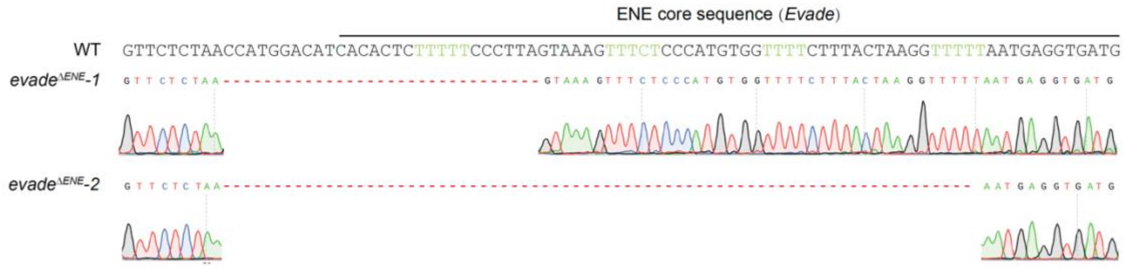

Fig. S3. Deletion of the ENE motif in *Evade*. *Evade* mutations deleted with the ENE motif were generated by the CRISPR-Cas9 approach. Chromatogram of the Sanger sequencing data is shown, confirming the large deletions detected in the *evade*$^{\Delta ENE}$*-1* and *evade*$^{\Delta ENE}$*-2* mutants.

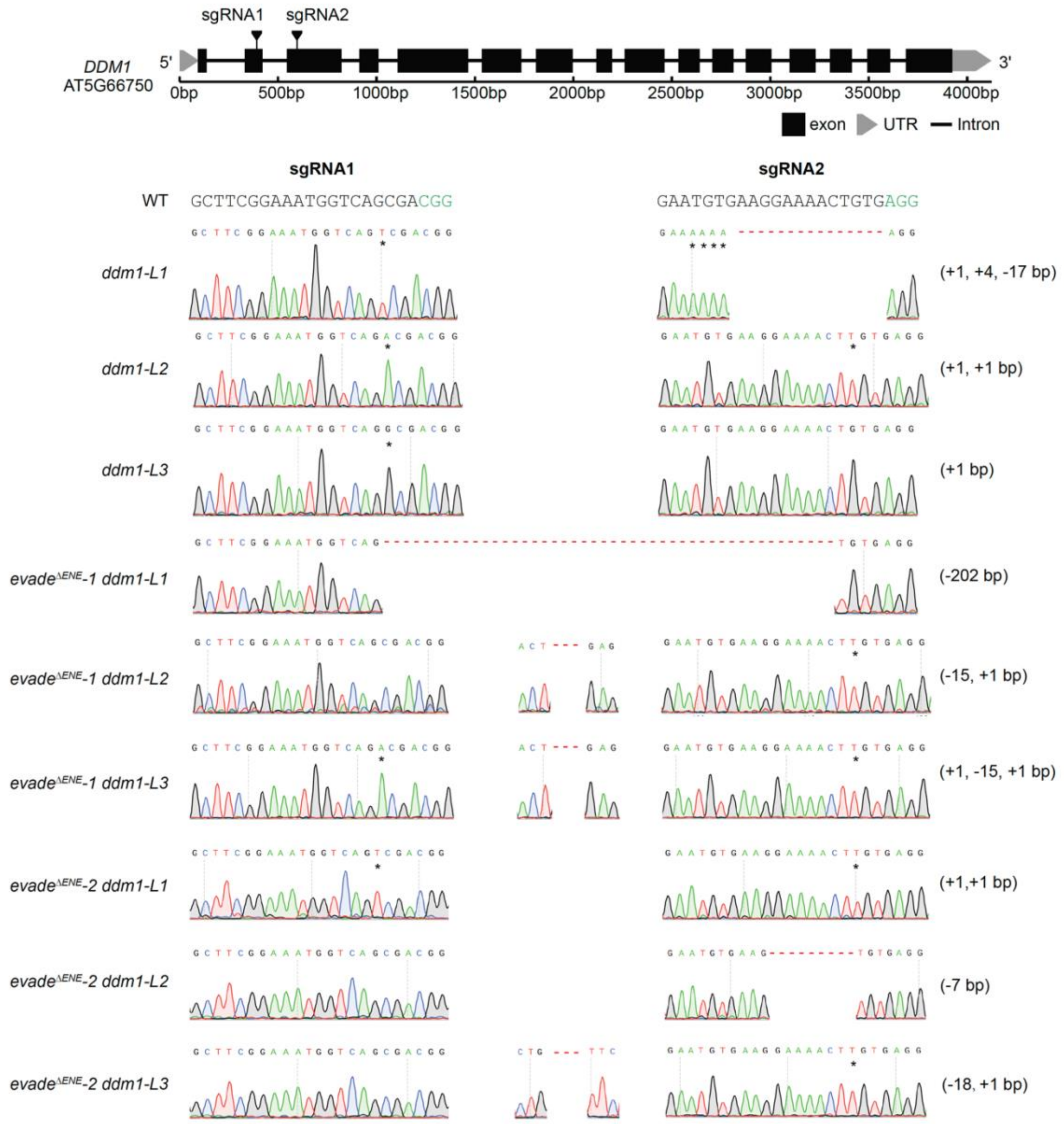

Fig. S4. *De novo* mutations of *DDM1*. *DDM1* mutations generated by CRISPR-Cas9 in the WT, *evade*$^{\Delta ENE}$*-1* and *evade*$^{\Delta ENE}$*-2*. Numbers in brackets indicate nucleotide insertions and deletions. The gene structure of *DDM1* and sgRNA-targeted regions are shown at the top.

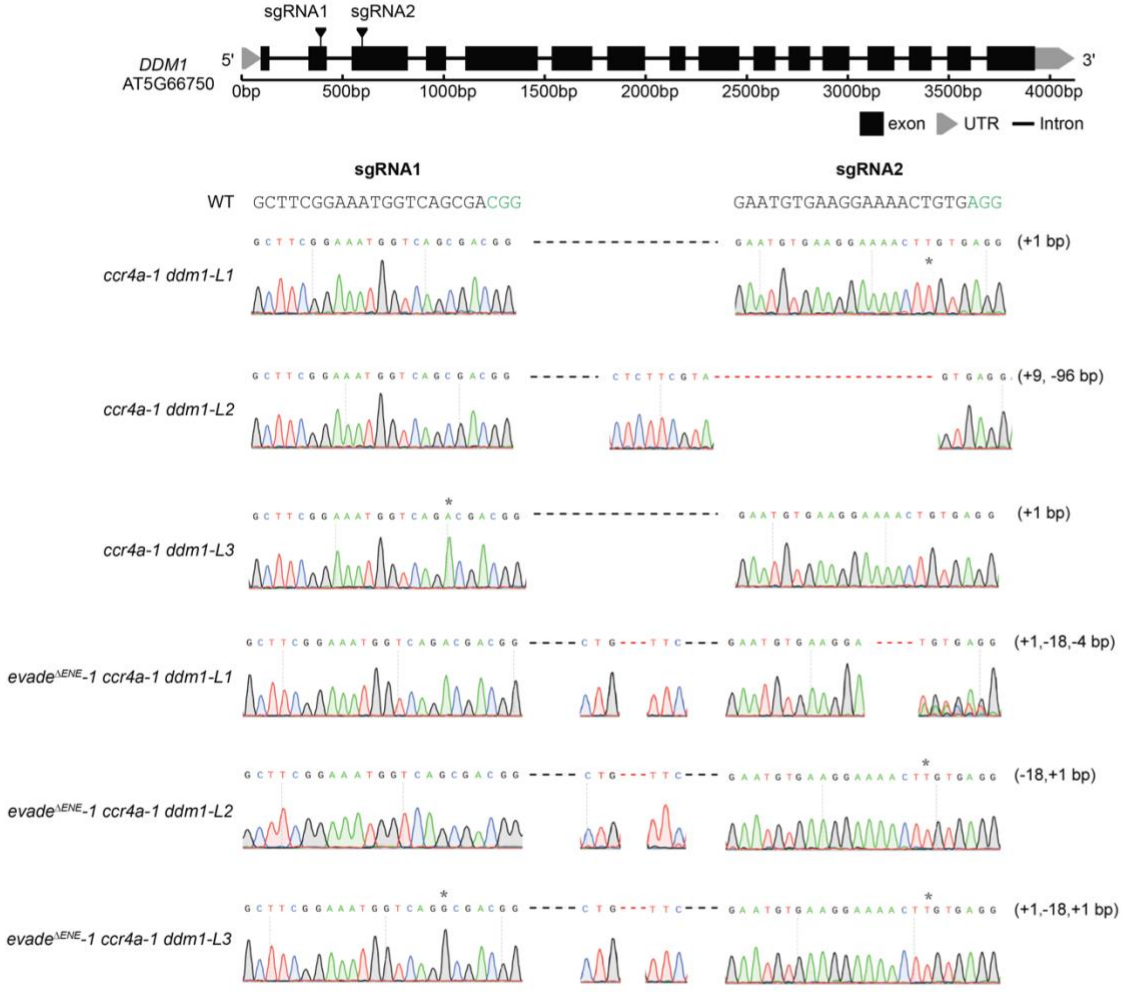

Fig. S5. *De novo* mutations of *DDM1*. *DDM1* mutations generated by CRISPR-Cas9 in *ccr4a-1* and *evade*$^{\Delta ENE}$*-1 ccr4a-1*. Numbers in brackets indicate nucleotide insertions and deletions.

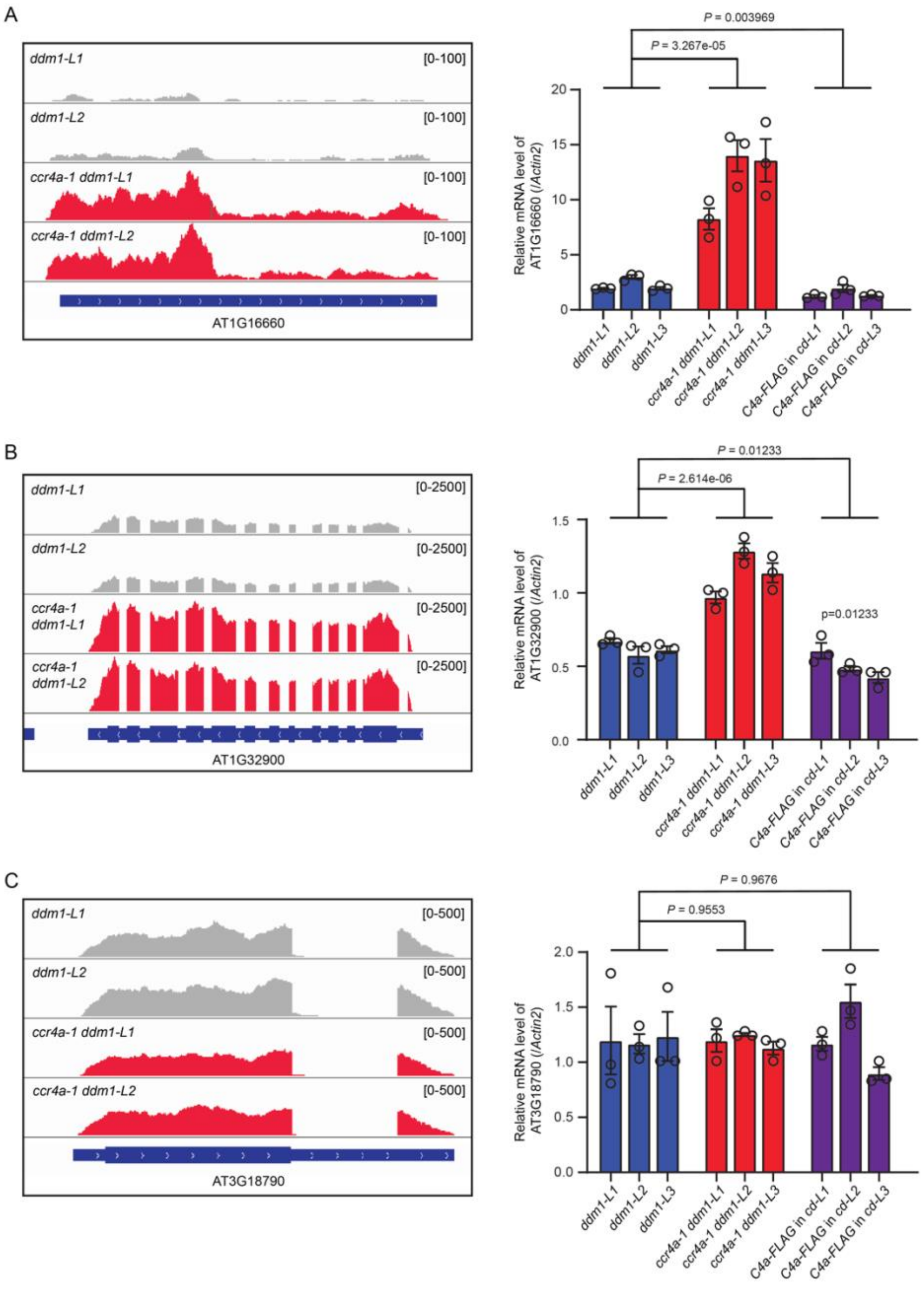

Fig. S6. CCR4a-regulated transcripts. (A-C) RNA-seq of *ddm1* and *ccr4a-1 ddm1* (left) and RT-qPCR of *ddm1*, *ccr4a-1 ddm1*, and *pCCR4a::CCR4a-FLAG* in *ccr4a-1 ddm1* (*C4a-FLAG* in *cd*) plants (right). AT1G16660 and AT1G32900 were chosen as CCR4a direct targets, and AT3G18790 as a negative control. RNA-seq data was retrieved from PRJNA940263. For RT-qPCR results, data are mean ± s.e.m of three biological repeats, and the raw data are shown as open circles. *P* values were obtained by the two-tailed Welch's t-test.

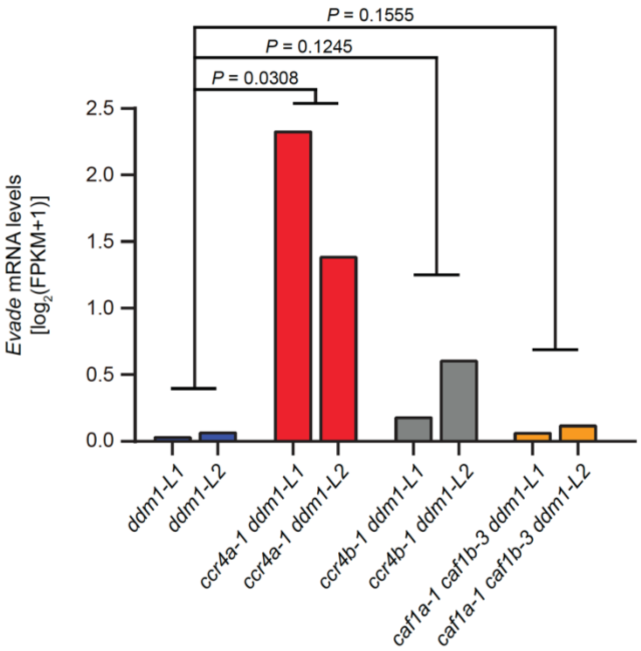

Fig. S7. *Evade* expression levels in mRNA deadenylase mutants. RNA-seq of *ddm1*, *ccr4a-1 ddm1*, *ccr4b-1 ddm1*, and *caf1a-1 caf1b-3 ddm1* mutants. RNA-seq data was retrieved from PRJNA940263. Log_2-converted FPKM at the *Evade* locus is shown. *P* values were obtained using two-tailed Welch’s t-test.

Table S1. Oligonucleotides used in this study.

Experiments	Name	Strand	Sequence (5’ -> 3’)
Genotyping	*ccr4a-1*	forward	TAAATCATTGGGCTGCTCATC
		reverse	AAACAACGGTTCACTTGATGC
	ccr4b-1	forward	TGTGTTGTGGCTAATGCTGAG
		reverse	TGTGTTGTGGCTAATGCTGAG
	caf1a-1	forward	AAAATTTCTTCTTTCCAAAGAACG
		reverse	GTTTCTCGTCCCCACTTCTTC
	caf1b-3	forward	CTAAGATCGATGAGAGCGTCG
		reverse	TTCAATTTCCGTGACTTCGAC
	evade	forward	GTGTTTTCCACGGGGTGTTC
		reverse1	CAAGTCTCAAAGTTTGCAACTTC
		reverse2	ATAACCGTTCCATAATCTACCGC
	ddm1	forward	TAAGTCCCTCCACCTTTCCTTT
		reverse	ACCCACAAGTTGTTCAAACTAAGAAT
RT-qPCR	*EF-1α*	forward	TGAGATGCACCACGAAGCTC
		reverse	CCAACATTGTCACCAGGAAGTG
	eGFP	forward	GAGCAAAGACCCCAACGA
		reverse	GTCCATGCCGAGAGTGAT
	Basta	forward	GCGACGAGCCAGGGATAG
		reverse	CTACATCGAGACAAGCACGG
	Actin2	forward	GGTAACATTGTGCTCAGTGGTGG
		reverse	CAACGACCTTAATCTTCATGCTGC
	EVADE	forward	AATCGAAAGGGGGAGAAAGA
		reverse	CGCAAAACAAATGTCAGGTC
ALE-qPCR	*PopRice* PCR	forward	TGTAGGAATAAGTCTTGGCTGG
		reverse	AGGAACTTGATCCTCCCAAG
	ALE adaptors	top	AGAGAGTAATACGACTCACTATAGGGACA CGACGCTCTTCCGATCT
		bottom	5'phosAGATCGGAAGAGCGTCGTGTCCCT ATAGTGAGTCGTATTACTCTCT
	ALE RT		AGACGTGTGCTCTTCCGATCTGCTCTGAT ACCA
	Evade qPCR	forward	TCCGATCTTATTGATCAAGAC
		reverse	AGACTTCTCATATGTTCGGC
	PopRice qPCR	forward	TCCGATCTTGTAGGAATAAGT
		reverse	GAGCATCCAAACTGAAAGTA

RIP-qPCR	Region A	forward	AATCGAAAGGGGGAGAAAGA
		reverse	CGCAAAACAAATGTCAGGTC
	Region B	forward	ATCTCACTCTTGATCTCATGTTCTC
		reverse	caagagtctaagcttggaacacatg
	AT1G16660	forward	ATCAAGGCCACCAGCTTACC
		reverse	GTAGGGGTCTGAGAGAGAGG
	AT1G32900	forward	AGCCGTGTGGTCTCATTCAG
		reverse	CAGTGTCCACAAGTCCACCA
	AT3G18790	forward	CGTTGAGCTAGGTGGTCACA
		reverse	TAACCAGGACCACGACCACT
ddPCR	*Evade*	forward	AGATTCTCCACGAAAGGCGT
		reverse	TAAGCAAGTGTTTAATTAGGTCATT
		probe	FAM-ACCCTGGATTTAAGGTGAGAAGGAGTC-BHQ3
	Actin2	forward	GCTGATGATATTCAACCAATCG
		reverse	CCTACCAACAACACTGGGAA
		probe	HEX-TGGTACCGGTATGGTGAAGGCTGG-BHQ3
dual-LUC	*CCR4a*-KpnI	forward	TTTGGAGAGGACAGGGTACCATGCTTAGCGTTATACGAGTGC
	CCR4a-XbaI	reverse	GAACTAGTGTCGACTCTAGACTATAAAATATTGTTTCGTCTGGCCC
	ENE	forward	TCTTGACTGACTGAGGATCTTCTCATGTTCTCTAACCATG
	ΔENE	forward	GGGATCTTGACTGACTGAGGATCTCCAAGCTTAGACTCTT
	3’LTR	reverse	CTTCATCTTCATATTCTAGTTGAAAGAATATGCGGAATTG
Thermal denaturation assay	ENE^{Evade}	forward	CATCACACTCTTTTTCCCTTAGTAAAGTTTCTCCCATGTGGTTTTCTTTACTAAGGTTTTTAATGAGGTGATG
	ENE^{Evade} Mut1	forward	CATCACACTCTTTTTCCCTTAGTAAAGTTTCTCCCATGTGGTTTTCTTTACTAAGGTTTTTAATCAGGTGATG
	ENE^{Evade} Mut2	forward	CATCACACTCCCCTTCCCTTAGTAAAGTTTCTCCCATGTGGTTTTTCTTTACTAAGGTTCCCAATGAGGTGATG
	ENE^{Evade} Mut3	forward	CATCACACTCTTTTTCCCTTAGTAAAGTTTCTCCCATGTGGTTTTTCTTTACTAAGCTTTTTAATGAGGTGATG
	ENE^{Evade} Mut4	forward	CATCACACTCTTTTTCCCTTAGTAAACTTTCTCCCATGTGGTTTTTCTTTACTAAGGTTTTTAATGAGGTGATG

ENE^{Evade} Mut5	forward	CATCACACTCTTTTTCCCTTAGTAAAGCCCCTCCCATGTGGTTCCCCTTTACTAAGGTTTTTAATGAGGTGATG
ENE^{Evade} Mut6	forward	CATCACACTCTTTTTCCCTTAGTAAAGTTTCTCCCATGTGCTTTTTCTTTACTAAGGTTTTTAATGAGGTGATG
ENE^{Evade} Mut7	forward	CATCACACTCTTTTTCCCTTAGTAAAGTTTCTCCCATGTGGTTTTTCTCTACTAAGGTTTTTAATGAGGTGATG
